# Editorial: Molecular Mechanism of Neuronal Dysfunction in the Diabetic Brain

**DOI:** 10.3389/fendo.2016.00045

**Published:** 2016-05-20

**Authors:** Marc Lee Goalstone, Subbiah Pugazhenthi

**Affiliations:** ^1^Denver VA Medical Center, Denver, CO, USA; ^2^University of Colorado School of Medicine, Aurora, CO, USA

**Keywords:** Alzheimer’s disease, diabetes, neuroinflammation, amyloid precursor protein, editorial

**The Editorial on the Research Topic**

**Molecular Mechanism of Neuronal Dysfunction in the Diabetic Brain**

Insulin signaling plays a significant role in the brain synaptic function. Insulin resistance is associated with reductions in cerebral glucose metabolism even in prediabetic individuals (1). Cognitive dysfunction in chronic diabetes is being recognized as a complication that needs to be seriously addressed. Epidemiological studies have also identified diabetes as a risk factor for Alzheimer’s disease (AD) (2, 3). The link between diabetes and AD is further supported by studies in animal models (3). Chronic hyperglycemia-induced accumulation of advanced glycation end products (AGEs) (4) and their receptor RAGE are among the mechanistic links between diabetes and AD (5). Soluble form of RAGE (sRAGE) acts as a decoy for RAGE and provides a counter-regulatory mechanism. Selvin et al. have reported that low levels of sRAGE increase the risk of diabetes and mortality (6). Decreased levels of sRAGE have been also observed in AD patients (7). In this special issue, six articles (three original reports and three reviews) highlight the interactions of these two aging-associated diseases from several angles.

van Dijk et al. describe AD as a complex, multifactorial disease that includes, but not limited to neuroinflammation, processing of amyloid precursor protein (APP) to amyloid β peptide, and tau protein hyperphosphorylation. The authors propose that these pathophysiologic mechanisms are propagated by obesity, metabolic syndrome, and type-2 diabetes mellitus. The authors assert that complex and interacting mechanisms are not yet completely understood and will require further analysis. This review discusses several of these interacting mechanisms in the animal models and compares them with clinical data, thus giving an overview about the current knowledge of diabetes and its possible relationship to AD. Vinik et al. have suggested that inflammatory cytokines as well as oxidative and nitrosative stress in diabetes play important roles in autonomic dysfunction (8). Pharmaceutical approaches suggested in this review article to restore autonomic function can also provide potential therapeutic strategies for targeting neurodegeneration in the brain.

Another review article by Sato and Morishita highlights the common brain pathologies of diabetes and AD. For example, diabetes is associated with reduced hippocampal volume due to neuronal loss and aberrant functional connectivity. Because insulin inhibits tau phosphorylation through negative regulation of GSK3β and protein phosphatase 2A, diabetes promotes the formation of neurofibrillary tangles as a result of tau hyperphosphorylation, an important pathological feature of AD. Defective brain glucose metabolism is another common feature of diabetes and AD because insulin signaling is altered by Aβ. Impaired cerebrovascular function in the diabetic brain can lead to blood–brain barrier (BBB) damage as in AD. Thus, diabetes can impair cognitive function by both Aβ/tau-dependent and -independent mechanisms.

In an intriguing report, van der Harg et al. highlight the association of diabetes, neuroinflammation, and AD. The authors examined whether neuroinflammation could be the mechanistic trigger to induce tau phosphorylation in the brain of diabetic animals. This study utilized two diabetic rat models, rats on a free-choice high-fat high-sugar (fcHFHS) diet that were insulin resistant and streptozotocin-treated rats that were insulin deficient. As expected, the streptozotocin-treated animals showed increased tau phosphorylation in the brain, whereas the fcHFHS diet-fed animals did not. Interestingly, neither of the diabetic animal models showed reactive microglia or increased GFAP and COX-2 levels, markers of inflammation, in the brain. These authors concluded that neuroinflammation is not the mechanism that explains the close connection between diabetes and AD in terms of Tau pathology.

Bartman et al. focus on another pathway involving glycogen synthase kinase-3, phosphatidylinositol 3-kinase (PI3K), and Wnt/β-catenin. Genetic deletion of both *Gsk-3*α and *Gsk-3*β in mouse embryonic stem cells (ESCs) appears to lead to the constitutive activation of the Wnt/β-catenin and insulin signaling pathways. This study compared the gene expression profiles in *Gsk-3*α^−/−^ *Gsk-3*β^−/−^ ESCs to mouse ESCs in which Wnt/β-catenin signaling or PI3K-dependent insulin signaling were constitutively active. Results showed that Wnt signaling had a greater effect on upregulated genes in the *Gsk-3*α^−/−^; *Gsk-3*β^−/−^ ESCs, whereas PI3K-dependent insulin signaling was more responsible for the downregulation of genes in the same cells.

Hypoglycemia is a known side effect of insulin therapy in both type-1 and type-2 diabetes. Recurrent hypoglycemic (RH) episodes are of concern in diabetic patients and are the biggest obstacle to intensive insulin therapy aimed at tight glycemic control. McNay’s report (McNay) examined the impact of RH on the amygdala, which plays a key role in anxiety and mood. Animals in the RH group showed heightened anxiety as measured by significantly less time spent in the open arms during plus-maze testing. This observation was also accompanied by increased norepinephrine release in the basolateral amygdala (BLA), a marker for amygdala cognitive modulation. These findings are consistent with the clinical experience of diabetic patients, especially with respect to impaired mood control and increased anxiety.

Sebastiao et al. highlight an important therapeutic angle in the diabetes/AD connection. Although the primary goal of diabetes treatment is achieving normoglycemia, these authors indicate that this is not sufficient because of the CNS changes in diabetic patients that could go unnoticed over decades. To normalize these defects, we need to take advantage of the beneficial actions of antidiabetic drugs in the brain. It has been suggested that therapeutic strategies against diabetes might be also beneficial against AD. Especially, glucagon-like peptide-1 (GLP-1)-based therapies have shown promise in the treatment of AD because GLP-1 can pass through BBB. Velmurugan et al. have previously reported the neuroprotective actions of GLP-1 in human neuroprogenitor cell-derived neurons (9).

The reports in this special issue are, but a few, of the enormous amount of investigation into the association between neuroinflammation, diabetes, and AD. The life expectancy of people with diabetes is steadily increasing with the availability of several classes of drugs for managing diabetes, its complications, and glycemic control. Dementia among diabetic patients will be a major health problem in the upcoming years with intellectually challenging life styles. Beneficial actions of antidiabetic drugs in the brain need to be considered in long-term diabetes management.

## Author Contributions

All authors listed have made substantial, direct, and intellectual contribution to the work and approved it for publication.

## Conflict of Interest Statement

The authors declare that the research was conducted in the absence of any commercial or financial relationships that could be construed as a potential conflict of interest.
